# Aspartyl protease MfSAP2 is a key virulence factor in mycelial form of skin fungi *Malassezia furfur*

**DOI:** 10.1042/BCJ20253109

**Published:** 2025-12-24

**Authors:** Wisely Chua, Yuanyuan Hei, Li Fang Koh, Belle Lin Hwee Yap, Hui Ling Saw, Thaís Helena Maciel Fernandes, Elany Barbosa da Silva, Shirlyn Goh, Thomas L. Dawson, Anthony J. O’Donoghue, John E. Common, Hao Li

**Affiliations:** 1 Molecular Engineering Lab, Institute of Molecular and Cell Biology, Agency for Science Technology and Research, 138673, Singapore; 2 Department of Chemistry, National University of Singapore, 3 Science Drive 3, Singapore 117543; 3 A*STAR Skin Research Labs (A*SRL), Agency for Science, Technology and Research (A*STAR), 8A Biomedical Grove, 138648Singapore; 4 Skin Research Institute of Singapore (SRIS), 8A Biomedical Grove, 138648, Singapore; 5 Skaggs School of Pharmacy and Pharmaceutical Sciences, University of California San Diego, La Jolla, California, 92093, U.S.A.; 6 Translational and Clinical Research Institute and NIHR Newcastle Biomedical Research Centre, Newcastle University, Newcastle upon Tyne, U.K.

**Keywords:** protease, skin barrier, skin microbiome, virulence factor

## Abstract

*Malassezia* is the dominant genus of fungi residing on human skin and is associated with both healthy skin and many dermatological conditions. Among these skin diseases, pityriasis versicolor (PV) has strong etiological connections with *Malassezia*. In the hyper- or hypo-pigmented scales of PV patients, *Malassezia* is enriched in its mycelial form, which is rarely present on healthy skin. How these *Malassezia* hyphae contribute to disease pathology in PV is unknown. In this study, we observed a distinct shift in the extracellular proteolytic activity when *Malassezia furfur* transitions from yeast to hyphae. We identified that the expression of the aspartyl protease MfSAP2 is dramatically up-regulated at both the mRNA and protein level when *M. furfur* is in the mycelial form. We determined the protease substrate specificity and observed that MfSAP2 can degrade corneodesmosome proteins, which are intercellular adhesive proteins between corneocytes in the stratum corneum. In a 3D human skin model with MfSAP2 treatment, we observed clear degradation of corneodesmosin, a component of the corneodesmosome. Taken together, our study demonstrates that a secreted protease is a key virulence factor associated with *M. furfur* mycelium and is potentially involved in the disease pathogenesis of PV.

## Introduction

*Malassezia* is the predominant genus of fungi that resides on human skin [1–3]. While generally regarded as a commensal, *Malassezia* has been associated with several cutaneous conditions [4–7]. Among these dermatological diseases, pityriasis versicolor (PV) has well-characterized etiological connections with this genus of fungi [8]. *Malassezia* are dimorphic fungi; they exist mostly in the yeast form but are able to transition to mycelial forms in conditions that are yet to be fully understood [4,9]. In PV lesions, *Malassezia* is enriched in the mycelial form [10] where prevalence of hyphae is close to 100% in these sites while prevalence of hyphae on skin sites from healthy individuals is very low (6–7%) [4,7].

PV is a noninflammatory skin condition characterized by scaly hyper- or hypopigmented plaques [11]. PV lesional sites have slight to moderate hyperkeratosis involving extensive basket-and-weave structure [12] in the upper stratum corneum (SC), the outermost layer of the epidermis comprising flattened, dead corneocytes. PV lesions are mainly found at the sebaceous skin sites such as the trunk, back, and facial area, and this corresponds to the higher abundance of the lipophilic *Malassezia* at these sites [13,14]. Histological studies on *Malassezia* at these sites revealed that the hyphae protrude into the SC but not deeper into the epidermis or dermis. This probably accounts for the lack of inflammation at the affected lesions.

One of the best-studied fungal examples of pathogenicity is *Candida albicans* hyphae [15,16]. In *C. albicans*, the hyphae penetrate the deeper regions of the epithelial layers of the mucosal surface, and a crucial agent facilitating this tissue invasion is a class of enzymes known as the secreted aspartyl proteases (Saps) [17]. *C. albicans* harbors 12 Saps, and Saps 4–6 are selectively expressed in the mycelial form to degrade connective tissue to enable deeper penetration of this pathogen [18–20]. Similarly, *Malassezia globosa* and *Malassezia furfur*, the two species of *Malassezia* that have been implicated in PV, contain 14 and 5 putative extracellular aspartyl proteases, respectively [21]. In our previous work, we focused on determining the functions of the dominant secreted aspartyl protease (SAP1) in the yeast form of both species. Our studies revealed that the *Malassezia* SAP1 can degrade adhesion-related proteins and extracellular matrices, which overall promotes a planktonic cellular state that can aid in fungal colonization and potentially contribute to inflammation at barrier-compromised skin sites [22–24].

At PV lesional sites, *M. globosa* was reported to be the predominant species present in hyphae form [12]; however, to date, no *M. globosa* strain has been successfully induced to form mycelium in laboratory culture. We instead turned to *M. furfur*, which has been reported to have increased prevalence at the lesional sites from PV patients in countries with tropical climates [11]. In this study, we show that aspartyl proteases dominated the *M. furfur* extracellular protease activity profile when hyphae were formed. Hyphae formation was sensitive to inhibition of aspartyl proteases, but not completely attenuated even in the absence of this protease activity. We show that the transition from yeast to hyphae was also accompanied by a distinct shift in the secreted protease expression and substrate cleavage profile and identified *M. furfur* SAP2 (MfSAP2) as a key enzyme produced in this transformation. We further observed that recombinant MfSAP2 degrades several key corneodesmosome proteins *in vitro*. In particular, we observed efficient cleavage of corneodesmosin (CDSN), an integral protein of the corneodesmosome complex important for skin barrier integrity. When full-thickness 3D skin cultures were treated with recombinant MfSAP2, we observed clear reduction in epidermal CDSN. Taken together, our study identifies an important virulence factor that is expressed specifically during the hyphae form of *Malassezia* and demonstrates that this enzyme has profound effects on structuring the stratum corneum during disease.

## Results

### *In vitro* mycelial induction optimization

**Figure 1:**
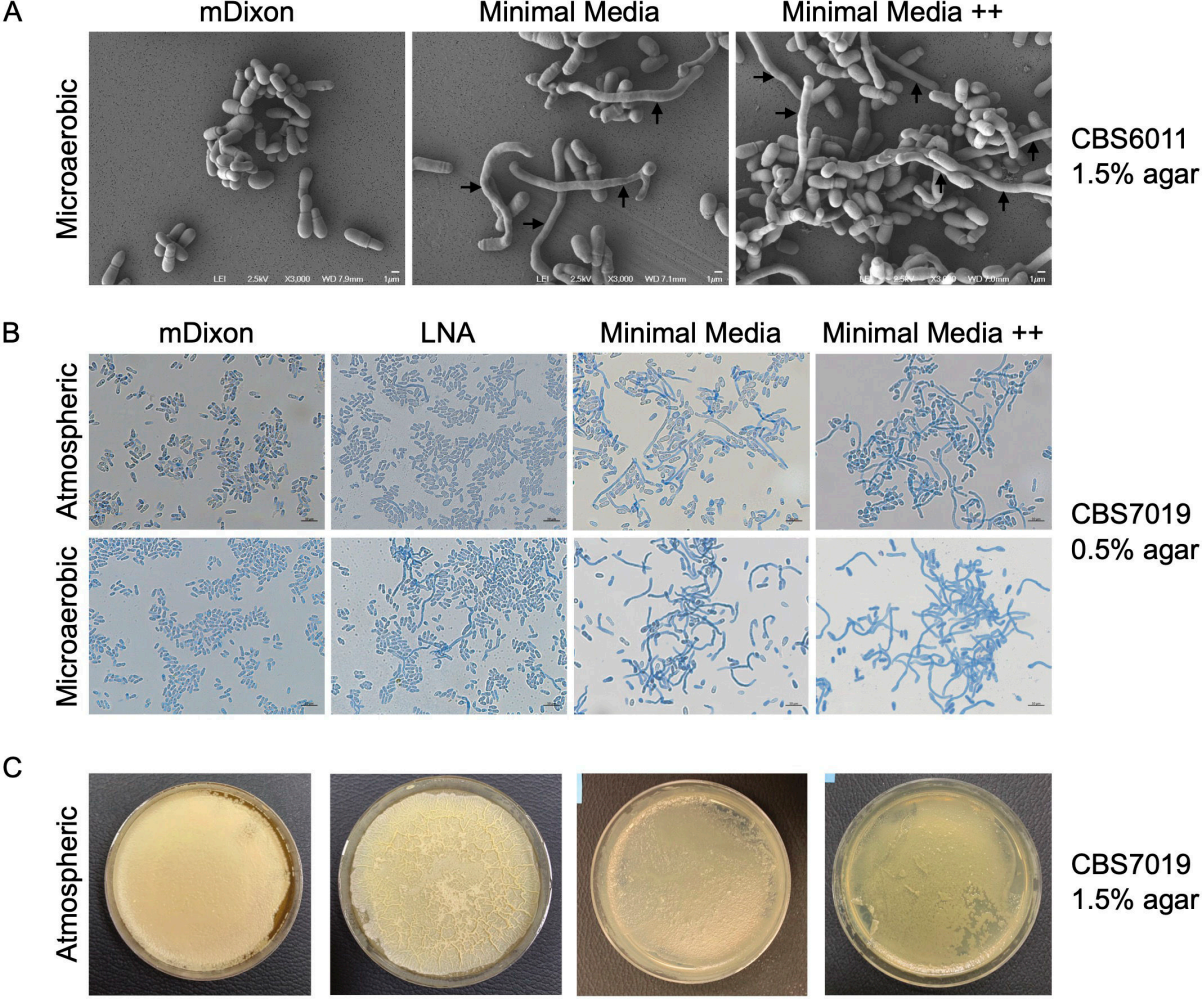
Optimization of culture conditions to induce *M. furfur* hyphae formation *in vitro*. **(A**) Scanning electron microscopy images of *M. furfur* CBS 6001 cultures on 1.5% mDixon (left), minimal media (center) or minimal media supplemented with kojic acid and L-DOPA (minimal media ++, right). All cultures were kept in microaerobic conditions. Hyhae are indicated by arrows. Scale bar represents 1 µm. (**B**) Light microscopy images of *M. furfur* CBS 7019 culture in 0.5% agar containing four different media. The upper panel shows cultures kept at atmospheric conditions and the lower panels show culture in microaerobic conditions. Scale bar represents 10 µm. (**C**) Picture of each *M. furfur* CBS 7019 agar culture plate kept at atmospheric conditions.

We first set out to optimize conditions to induce *M. furfur* mycelium production using a previously reported minimal culture medium (MM) grown as planktonic, 0.5% or 1.5% agar culture [25]. L-DOPA and kojic acid (MM++) were further supplemented to enhance hyphae growth [25]. This was compared with *M. furfur* culture in two rich media—modified Dixon media (mDixon) and a modified LNA media containing olive oil (mLNA). The cultures were then incubated under aerobic (atmospheric) or microaerobic conditions—another condition previously reported to promote mycelium formation [25]. We observed that hyphae formation is highly dependent on the *M. furfur* strain; CBS 14141 and CBS 1878 remained in the yeast form under all conditions, while CBS 6000, 6001, and 7019 were able to form hyphae (Figure 1A and B and Table 1) in several culture conditions. Hyphae formation cannot be induced in trans, as addition of the extracellular media of mycelial forming cultures to CBS 14141 did not result in formation of mycelium (Supplementary Figure S1). Consistent with previous reports, hyphae formation was more prevalent and longer filaments were observed when cultured in 0.5% agar, and this enhancement was especially significant under aerobic conditions [25]. While both MM and MM++ media contain minimal nutrients for growth, we observed that nutrient deprivation is not a necessary factor for hyphae formation. All strains capable of forming hyphae produced mycelium in the rich mLNA media, and this was accompanied by the appearance of a wrinkled surface when cultured on 1.5% mLNA agar (Figure 1C). Furthermore, mycelial formation is reversible—the hyphae transitioned back to the yeast form when the induction media was changed to mDixon (Supplementary Figure S1). We subjected several *M. globosa* strains (CSB 7874, CBS 7966, and CBS 7990) and *M. sympodialis* ATCC 42132 to the same induction conditions, but no hyphae were observed for all these strains (Table 1).

**Table 1:** Hyphal induction of *Malassezia* species and strains at different conditions

1.5% agar		Aerobic				Microaerobic			
		mDixon	MM	MMK	mLNA	mDixon	MM	MMK	mLNA
*M. furfur*	CBS 14141	-	-	-	-	-	-	-	-
	CBS 1878	-	-	-	-	-	-	-	-
	CBS 6000	-	-	-	++	+	+++++	+++	+
	CBS 6001	-	-	+	++++	-	++++	+++	+++++
	CBS 7019	+	++	+++	+++	-	++++	+++++	++
*M. globosa*	CBS 7874	-	-	-	-	-	-	-	-
	CBS 7966	-	-	-	-	-	-	-	-
	CBS 7990	-	-	-	-	-	-	-	-
**0.5% agar**		**Aerobic**				**Microaerobic**			
		**mDixon**	**MM**	**MMK**	**mLNA**	**mDixon**	**MM**	**MMK**	**mLNA**
*M. furfur*	CBS 14141	-	-	-	-	-	-	-	-
	CBS 1878	-	-	-	-	-	-	-	-
	CBS 6000	-	+++++	+++	++++	-	++++	++++	+++
	CBS 6001	-	+++	+++	++++	-	++++	++	+++
	CBS 7019	-	+++++	++++	+++	-	++++	+++++	+++
*M. globosa*	CBS 7874	-	-	-	-	-	-	-	-
	CBS 7966	-	-	-	-	-	-	-	-
	CBS 7990	-	-	-	-	-	-	-	-
*M. sympodialis*	CBS 42132	-	-	-	-	-	-	-	-
Rank	−	+	++	+++		++++		+++++	
Approximate %	0%	<1%	1-5%	6-15%		15-30%		30-50%	

### Extracellular protease activity of *M. furfur* hyphae is distinct from that of yeast culture

To quantify protease activities secreted into the extracellular media during hyphae formation, we used a panel of 19 quenched fluorogenic substrates containing diverse peptide sequences (see Supplementary Table S2). CBS 7019 strain cultured in 0.5% MM++ agar was used to generate conditioned media as it had one of the highest mycelium formations (Table 1). When the media was assayed at pH 4.2, we observed robust substrate cleavage for substrates S5, S6, S10, and S11, and low cleavage of S1, S8, and S19 (Supplementary Figure S2a). None of the other substrates were cleaved. When the same media was assayed at pH 6.0, activity with S5, S6, and S10 was reduced by 1.5 to 5-fold while S11 remained unchanged. No activity was detected with any of the 19 substrates when media was assayed at pH 7.4 (Supplementary Figure S2a). These data reveal the proteases secreted by *M. furfur* are optimally active at acidic pH.

**Figure 2:**
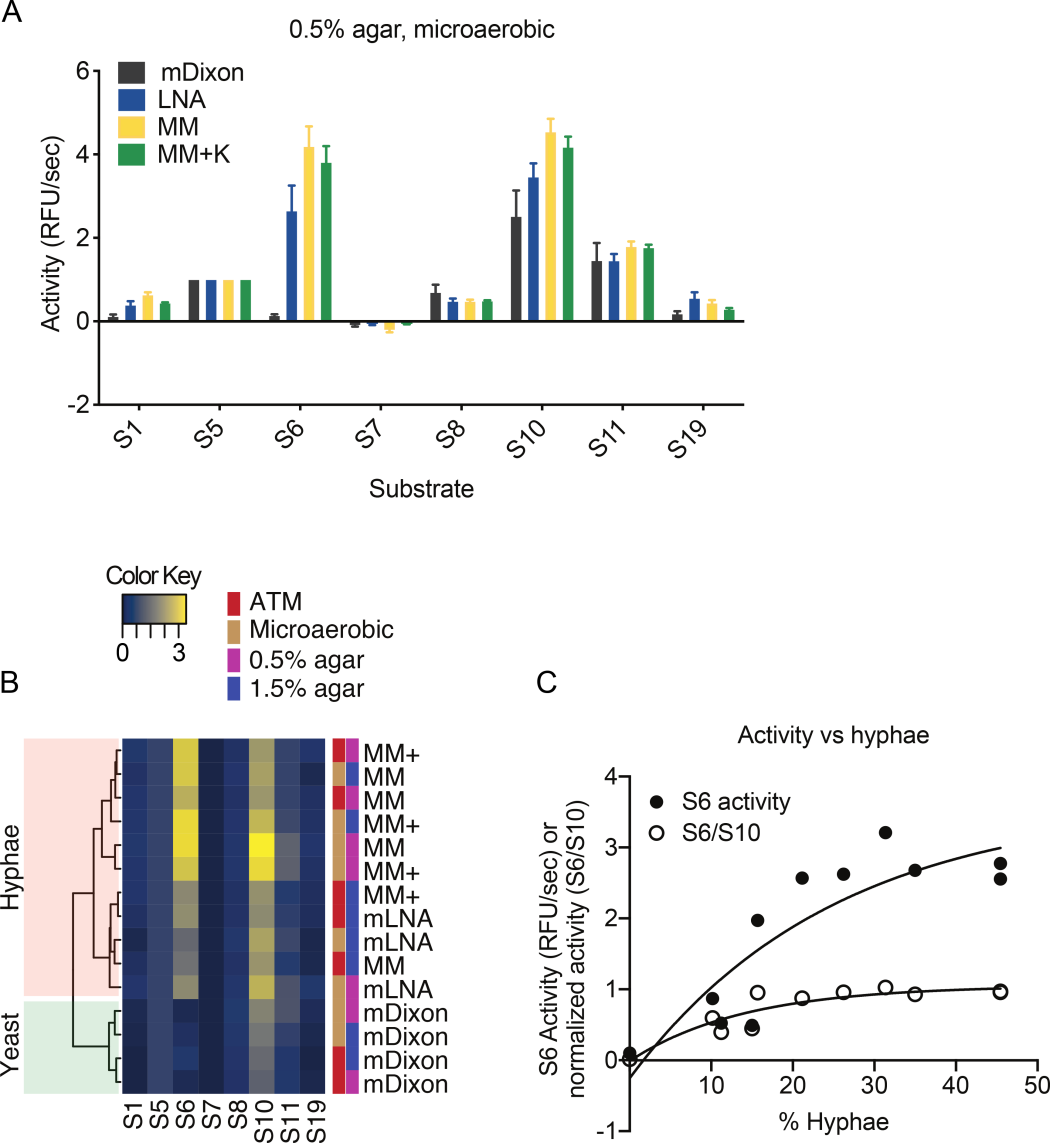
Extracellular protease activity in hyphae-containing cultures is distinct from yeast culture. **(A**) Proteolytic activity of culture supernatant isolated from *M. furfur* CBS 7019 agar cultures grown in 4 different media in microaerobic condition. Protease activity was assessed using internally quenched fluorescent substrates. (**B**) Heatmap of extracellular protease activity of *M. furfur* CBS 7019 cultures. The cultures were kept at either atmospheric or microaerobic conditions, either 0.5% or 1.5% agar in four different media. Protease activity of each substrate is normalized to S5 activity. Heatmap and hierarchical clustering are performed using R studio version 1.2.5042. (**C**) Correlation between protease cleavage of substrate S6 and percentage of hyphae in *M. furfur* CBS 7019 cultured in minimal broth media and incubated at 32°C at atmospheric conditions.

We next compared the extracellular protease activity of CBS 7019 cultured in yeast or hyphae-promoting conditions at pH 4.2 using only the fluorogenic substrates that were cleaved by proteases in the 0.5% MM++condition media (Figure 2A). We observed the secreted protease activity of hyphae-forming cultures was distinct from that of yeast cultures. Hierarchical clustering of the substrate cleavage profile at each culture condition shows that the secreted protease activity can distinguish if the culture is forming hyphae (Figure 2B). This was driven by increased processing of S6 and S10 substrates. In particular, cleavage of S6 was mostly absent in yeast culture but is robust in cultures where hyphae were formed (Figure 2B). Furthermore, cleavage of this substrate increased with increasing number of hyphae in the cultures, but this effect plateaued off after about 20% of hyphae were formed (Figure 2C).

### Secreted aspartyl proteases dominate protease activity of extracellular media but are not necessary for mycelial formation

**Figure 3:**
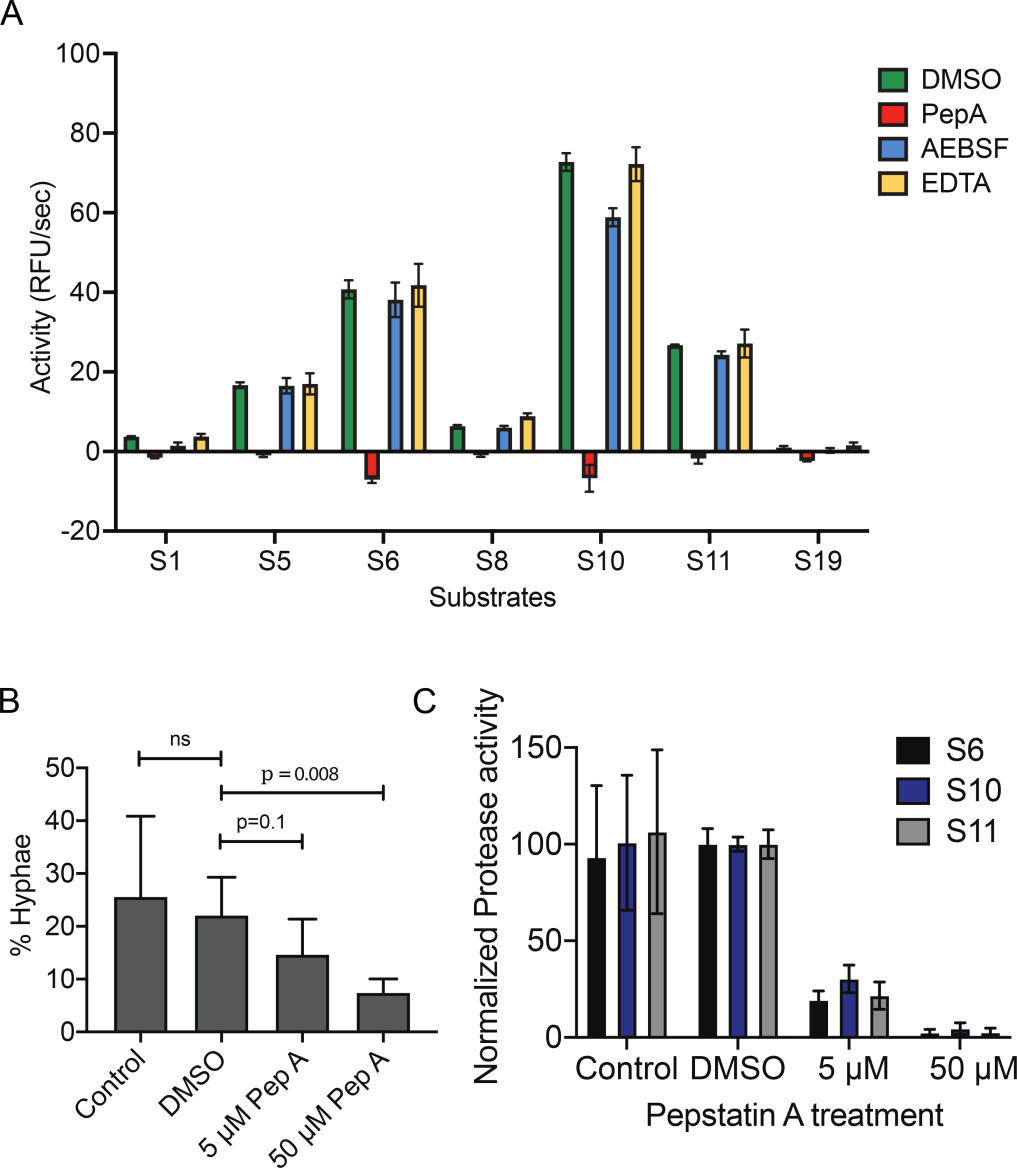
Aspartyl proteases are involved in hyphae formation. **(A**) Inhibition of extracellular protease activity using class-specific protease inhibitors. Culture supernatant from *M. furfur* CBS 7019 cultured in 0.5% agar, MM ++ media at microaerobic condition was treated with either DMSO, aspartyl protease inhibitor pepstatin A (50 µM), serine protease inhibitor AEBSF (2 mM) or metalloprotease inhibitor EDTA (10 mM) and the residual proteolytic activity was assayed using the indicated fluorescent substrates. *N* = 3 technical replicates. (**B**) Effect on % hyphae formation when control, DMSO or pepstatin A (5 and 50 µM) were added to broth *M. furfur* CBS 7019 cultures. The extracellular protease activities of these cultures were determined using fluorescent substrates in (**C**). *P*>0.05 for all substrates when comparing control with DMSO, and *P*<0.05 when comparing all substrates at 5 uM and 50 uM treatment to control (using Mann-Whitney test). *N* = 3 biological replicates. Error bars represent S.D.

*M. furfur* has a total of 14 predicted secretory proteases (5 aspartyl, 2 metallo, and 7 serine proteases). Using class-specific protease inhibitors, we observed that the extracellular protease activity was only inhibited by the aspartyl protease inhibitor pepstatin A (Figure 3A). This, along with the acidic pH 4.2 optimal for the extracellular protease activities (Supplementary Figure S2), suggests that aspartyl proteases account for the majority of the secreted protease profile. To assess if inhibition of secreted aspartyl protease has an effect on hyphae formation, we added pepstatin A to the culture at the start of induction and quantified hyphae formation. We observed that 5 μM pepstatin A significantly reduced (Figure 3B) mycelial formation. At 50 µM pepstatin A, where all aspartyl protease activities were inhibited (Figure 3C), mycelial formation was significantly reduced, but we were still able to detect a substantial amount of mycelium in culture. As pepstatin A did not affect viability of *M. furfur* up to 100 µM (Supplementary Figure S2), this suggests that aspartyl proteases produced during yeast to hyphae transition are involved but not necessary for this transformation.

### MfSAP2 is highly up-regulated when *M. furfur* transitions to hyphae

**Figure 4:**
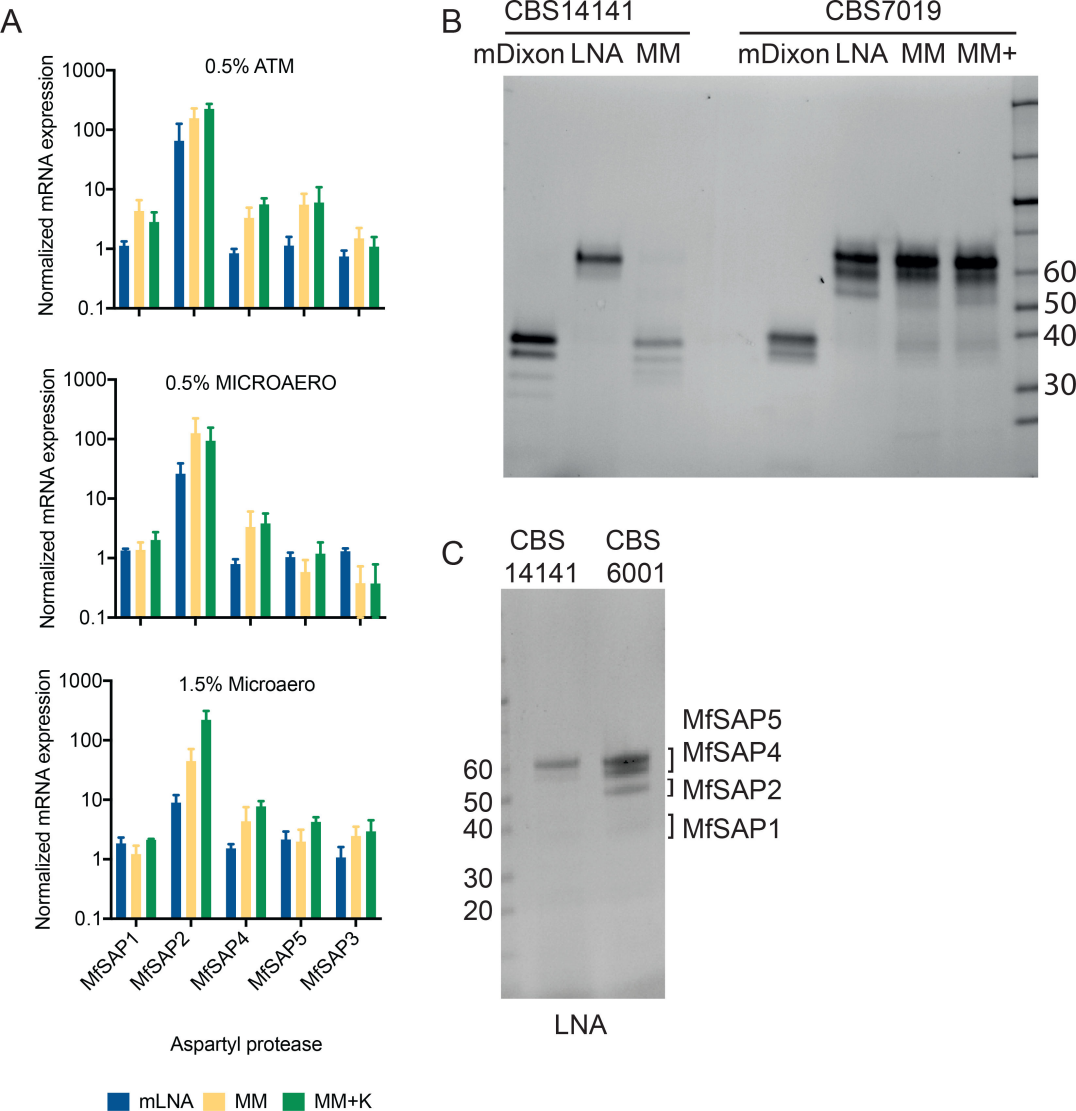
Expression of *M. furfur* aspartyl protease FUN_000222 (MfSAP2) is highly up-regulated during mycelial formation. **(A**) mRNA expression of the 5 extracellular aspartyl proteases in *M. furfur* CBS 7019 cultured in different conditions. Expression of each protease is first normalized to the housekeeping gene (actin) and then normalized to the control culture condition (mDixon). *N* = 3 biological replicates for each condition. Error bars represent S.D. (**B**) Aspartyl protease expression of the mycelial-forming CBS 7019 and the non-mycelial forming CBS 14141. Aspartyl proteases are first enriched from each culture media using pepstatin-agarose and the isolated proteins are ran on SDS-PAGE. (**C**) Comparison of the enriched aspartyl proteases in LNA media for CBS 14141 and CBS 6001 (mycelial-forming). Each of the protein bands are subjected to in-gel digestion followed by mass spectrometry and the identified proteases are indicated.

To assess which aspartyl protease expression accounts for the change in proteolytic activity during the yeast to hyphae transition, we assessed the mRNA expression of each secreted *M. furfur* CBS 7019 protease gene (*MfSAP1 - MfSAP5*) after 3 days in the mycelial induction media and compared this with the yeast culture in mDixon. In all conditions where hyphae were formed, we observed a dramatic up-regulation of *MfSAP2* (∼10–300-fold) (Figure 4A). While up-regulation of *MfSAP4* and *MfSAP5* was also observed to some extent, the increased expression of *MfSAP2* was especially significant given that it was barely detectable in the yeast form. To further assess the change in aspartyl protease expression at the protein level, we used pepstatin A-agarose for chemical affinity enrichment of the secreted aspartyl proteases [23] in the nonmycelial forming strain CBS 14141 and compared this with the mycelial forming CBS 7019 and CBS 6001 (Figure 4B). In CBS 14141, where no hyphae formed in all culture conditions, the secreted aspartyl protease profile was similar in mDixon and MM media but changed significantly when cultured in mLNA. In CBS 7019, which formed hyphae in mLNA, MM, and MM+ media, we observed a distinct shift in aspartyl protease expression compared with the yeast culture in mDixon. In the hyphae-forming cultures, the abundance of MfSAP1 (the two protein bands around 38 kDa) decreased dramatically, and instead, three high molecular weight proteins (50–60 kDa) were isolated. Based on molecular weight, these three proteins were likely MfSAP2, 4, and 5. As mLNA media alone can result in a shift in aspartyl protease expression, we compared the pepstatin A-agarose enriched aspartyl proteases from CBS 14141 and CBS 6001 cultured in mLNA using SDS-PAGE followed by in-gel digest and mass spectrometry of each enriched protein band (Figure 4C). In both CBS 14141 and CBS 6001, we identified the two bands around 60 kDa to be MfSAP4 and 5. MfSAP2 (∼52 kDa) was only isolated in CBS 6001 but not CBS 14141 even though both strains possess the *MfSAP2* gene. Taken together with the qPCR expression analysis, we reasoned that MfSAP2 is expressed only in the mycelium form of *M. furfur*.

### MfSAP2 degrades key corneodesmosome proteins

**Figure 5:**
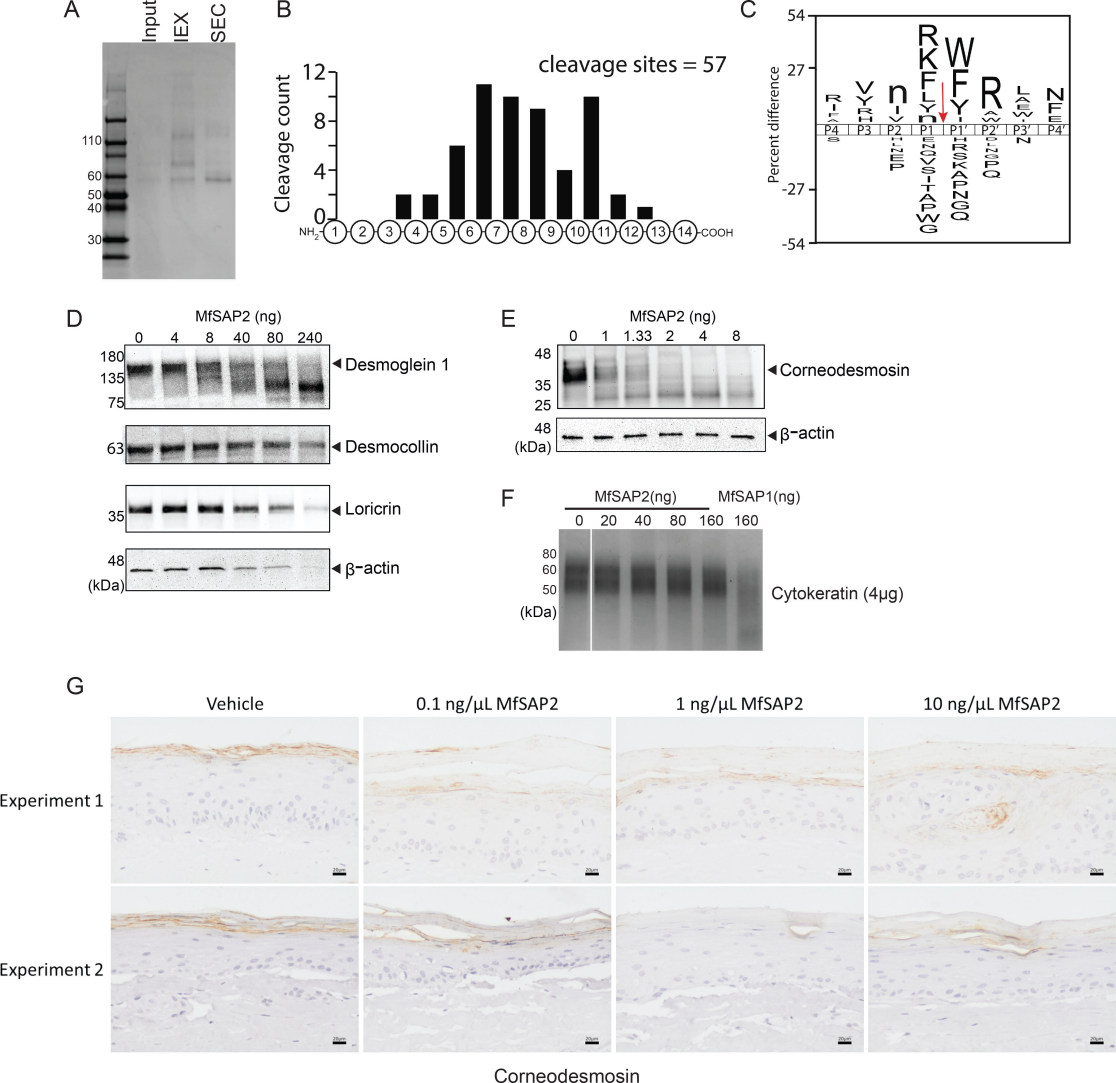
Recombinant expression of MfSAP2 reveals its role in degradation of corneodesmosome proteins. **(A**) Recombinant expression and purification of MfSAP2 in *Pichia pastoris*. Culture supernatant (input) was concentrated and purified first on cation exchange column (IEX) followed by size exclusion chromatography (SEC). (**B**) Cleavages detected at each amino acid position when recombinant MfSAP2 (rMfSAP2) was incubated with a pool of synthetic 14-mer peptides. Cleavages were monitored on LC-MS. (**C**) Substrate cleavage profile represented by iceLogo of the recombinant enzyme. The site of protease cleavage is indicated by the red arrow. (**D, E, and F**) Degradation of human skin epidermal proteins *in vitro*. Whole human skin epidermis (20 µg) was lysed and treated with different concentrations of rMfSAP2. The skin proteins were detected using western blot. β-actin was used as the loading control. (**G**) MfSAP2 degradation of cornedesmosin in 3D human skin equivalents. rMfSAP2 was applied on the skin cultures at three concentrations for 24 hr and harvested. Histology sections were obtained and immunohistochemistry was performed using antibodies directed against CDSN. Scale bar represents 20 µm.

To determine the function of the hyphae-specific MfSAP2, we first expressed and purified recombinant MfSAP2 (rMfSAP2) in *Pichia pastoris* (Figure 5A). We performed the protease cleavage assay with the same panel of fluorogenic substrates used to determine the total extracellular protease activity of *M. furfur* CBS 7019 in hyphae-promoting conditions and observed that the substrate cleavage profile of MfSAP2 closely follows that of the total extracellular media under hyphae-promoting conditions (Figure 2 and Supplementary Figure S4). To compare the substrate cleavage specificity of MfSAP2 to the previously characterized MfSAP1, we incubated 0.1 μg/ml of rMfSAP2 with a library of 228 synthetic peptide substrates containing diverse sequences spanning 14 residues [26]. Using LC-MS/MS to monitor the cleavage events, we generated a substrate specificity profile from the 57 cleavages by MfSAP2 across P4-P4′ (Figure 5B). Similar to MfSAP1 [22] and other fungal aspartyl proteases [27], this protease has a preference for cleavage at the C-terminal side of Arg, Lys, and Phe and N-terminal side of Trp, Phe, and Tyr (Figure 5C). Remarkably, MfSAP2 has a strong preference for Arg at the P2′ position, which is not observed in MfSAP1 [24]. This profile accounts for the high activity against the fluorogenic substrate SUBS017, which contains the Phe-Phe-Arg sequence.

In PV, the hyphae do not penetrate beyond the stratum corneum (SC) even though increased fragility of the SC was observed. Therefore, we reasoned that the physiological substrates of MfSAP2 are most likely SC proteins. Corneodesmosomes, which are protein complexes involved in interlocking individual corneocytes together, are key players controlling the formation of this SC pattern [28]. The extracellular motif of corneodesmosome comprises desmoglein 1 (DSG1), desmocollin 1 (DSC1), and corneodesmosin (CDSN) [29]. To assess the effect of recombinant MfSAP2 on SC proteins, we incubated the enzyme with cell lysate of human epidermis derived from human skin surgical waste and performed Western blot to determine the effect on SC proteins. We observed specific degradation of two key corneodesmosome proteins—DSG-1 and CDSN, with MfSAP2 having strong activity against CDSN even at a low 1:20,000 enzyme to substrate ratio (Figure 5D and E). Degradation of the two most abundant SC proteins, desmocollin and loricrin, was observed only at protease concentrations where the loading control actin was also degraded, likely due to non-specific degradation. In contrast, DSG1 and CDSN were processed by MfSAP2 at enzyme concentrations where little to no degradation of actin was observed, indicating that this cleavage is specific (Figure 5D). We then assessed MfSAP2’s activity against proteins found in the deeper epidermal and dermis layer by incubating MfSAP2 with purified forms of fibronectin, vitronectin, cytokeratin, and heat-denatured collagen (Supplementary Figure S4). While MfSAP2 can cleave these substrates *in vitro*, we observed that its activity toward these substrates is lower than that of the previously characterized MfSAP1. This is especially prominent for cytokeratin, where MfSAP2 only has weak activity at enzyme concentration where MfSAP1 can completely degrade cytokeratin (Figure 5F). This suggests that MfSAP2’s physiological substrates are likely localized to the SC.

To further investigate the activity of MfSAP2 on human skin, we utilized a full-thickness 3D reconstructed skin model consisting of human skin-derived keratinocytes grown over dermal fibroblasts to create a mature epidermis. We applied rMfSAP2 at three different concentrations onto the apical surface of the 3D skin for 24 h to determine its effect on the degradation of endogenous CDSN using immunohistochemistry. We observed significant degradation of CDSN even at a low concentration of 0.1 μg/ml of MfSAP2 (Figure 5G). However, no gross morphological changes were observed even at high concentration of MfSAP2, indicating that the effect of this protease is mostly localized to the stratum corneum.

## Discussion

*Malassezia* spp. have a crucial role in driving disease pathogenesis in the skin condition PV. However, the molecular factors that cause disruptions to the skin environment during PV are poorly characterized. In this work, we first optimized *in vitro* conditions that induce the yeast form of *M. furfur* to transition to hyphae, and this enabled us to study the extracellular proteases expressed in this process. We determined that the secreted protease expression changes dramatically during hyphae induction, with a significant up-regulation of the aspartyl protease MfSAP2. We further observed that MfSAP2 is a strong degrader of corneodesmosome proteins that function to link corneocytes for intact stratum corneum integrity. In particular, CDSN degradation was observed both *in vitro* and in full-thickness 3D skin culture when treated with MfSAP2. Overall, this study demonstrates that *Malassezia* extracellular aspartyl proteases are likely virulence factors driving PV pathogenesis.

While *Malassezia* hyphae have long been identified in skin samples, especially those isolated from PV patients, what triggers the transition from yeast to hyphae *in vivo* is still largely unknown. *In vitro*, a combination of reduced nutrient in culture media, lower oxygen levels, and density of agar culture can result in robust filament formation in *M. furfur*. As hyphae formation is highly strain specific as shown in this and previous studies [25], there are uncharacterized key genomic elements that control this morphological switch. In our inhibitor study, we determined that complete attenuation of secreted aspartyl protease activity does not lead to total inhibition of hyphae formation. This suggests that the change in *M. furfur* aspartyl protease expression, as characterized by MfSAP2 up-regulation, is a result but not cause of this morphological switch.

Our biochemical characterization of MfSAP2 indicates that this protease likely has an expanded substrate repertoire compared with the previously characterized MfSAP1 [22,24]. This is particularly evident from the degradation of the synthetic fluorogenic substrates, where MfSAP1 and MfSAP2 both strongly degrade S5 (MMPS024) and S10 (MMPS026) but S6 (SUBS017) is only cleaved by MfSAP2. Our substrate profile using the pool of tetradeca-peptides further confirmed that the affinity for SUBS017 is due to a preference of Phe, Phe, and Arg at the P1, P1′, and P2′ positions, respectively. We hypothesize that MfSAP2’s substrate profile enables this protease to access substrates in the stratum corneum, where *Malassezia* skin colonization occurs. Unlike the hyphae in *C. albicans* that penetrate through the epithelium, *Malassezia* hyphae are localized to the stratum corneum and do not penetrate the lower epidermis. As such, we reasoned that MfSAP2’s endogenous substrates are stratum corneum proteins and observed strong degradation of corneodesmosome proteins, in particular CDSN. As corneodesmosomes are essential in interlocking individual corneocytes together [28], the degradation of this important junction complex is tightly regulated and alterations to this process can lead to disruption in skin barrier integrity. Our data suggest that the extensive basket-and-weave formation [12] in the stratum corneum of PV lesional sites is due to the altered degradation of the corneodesmosome complexes.

Finally, while we have defined the switch in aspartyl protease expression in *M. furfur* during hyphae transition, determining this in *M. globosa* remains challenging. There is currently no reported *in vitro* condition that allows robust culture of *M. globosa* hyphae, and identifying such conditions will be crucial to enable further characterization of the secreted proteases in *M. globosa* hyphae. By sequence alignment, the *M. globosa* protease that has the highest sequence similarity to MfSAP2 is the previously characterized MgSAP1. This *M. globosa* protease is also MfSAP1’s closest homolog [24]. As such, it is not immediately apparent what MfSAP2’s homolog is in *M. globosa*. We are currently investigating expression levels of *M. globosa* aspartyl proteases in PV lesional sites in comparison with healthy skin sites to determine whether specific proteases are up-regulated in disease.

Overall, we have identified and functionally characterized a crucial *M. furfur* virulence factor that likely drives disease pathogenesis in PV. This protease is expressed only in the mycelium form of *M. furfur* and can degrade skin proteins that lead to poor skin integrity. While inhibiting this protease does not reverse mycelial formation, lowering the protease activity can reduce the amount of hyphae and likely slows progression of PV. As such, MfSAP2 can be a potential therapeutic target to alleviate PV symptoms.

## Materials and methods

### *Malassezia* strains and general culture conditions

*M. globosa* CBS 7966 and *M. sympodialis* ATCC 42132 were obtained from ATCC. *M. furfur* CBS14141 (JLPK23), CBS 6001, and CBS 7019 were obtained from Thomas Dawson at A*STAR Skin Research Laboratories. Routine culture of *Malassezia* strains was performed as indicated in protocols.io (https://dx.doi.org/10.17504/protocols.io.bjrakm2e).

### Culture mycelial induction

For *Malassezia* mycelial induction, three different media were prepared. Modified Leeming & Notman (LNA) media was prepared according to Kim et al. [30]. The two minimal media, MM and MM+, were prepared according to Youngchim et al. [25]. The MM media contains 15 mM glucose, 10 mM ammonium chloride, 50 mM glycine, 10 mM magnesium sulfate, 29.4 mM potassium phosphate, 3 µM thiamine, 0.1% Tween 40, and 0.1% Tween 80. The MM+ media contains the same components as the MM media with the addition of 1 mg/ml of kojic acid. mDixon media was prepared with 3.6% malt extract (Difco), 2% desiccated oxbile (Sigma-Aldrich), 0.6% peptone (Difco), 1% Tween 40 (Sigma-Aldrich), 0.2% glycerol (Sigma-Aldrich), and 0.2% oleic acid (VWR Chemicals). 0.5% or 1.5% (w/v) of Bacto agar (Becton Dickinson) was added to the media before autoclaving if required.

To induce hyphae formation, 120 µl of 10^8^ cells/ml of *Malassezia* culture plated on mDixon agar 48–72 h before induction were added to 12 ml of the induction media as described above. If the 1.5% agar-based media were used, the culture was spread gently on the agar and dried for 30 min in the biosafety cabinet. Cultures grown under microaerobic or anaerobic conditions were grown in AnaeroPack 2.5 l rectangular jars (Thermo Scientific) with Mitsubishi AnaeroPack-MicroAero 2.5 l (Thermo Scientific) or Oxoid Anaerogen 2.5 l packs (Thermo Scientific) added to generate each respective atmospheric condition.

All cultures were incubated for 96 h at 32°C. To check for hyphae growth by microscopy, cultures were first stained with Trypan Blue (ThermoFisher Scientific) for clarity. For the semi-quantification of hyphae growth in each culture, at least 10 microscopic fields per slide containing a total of 50–200 *Malassezia* cells were counted manually. The percentage of hyphae was calculated by dividing the number of hyphae in each view by the total number of cells.

### Gene expression analysis

For RNA extraction, *Malassezia* culture pellets were thawed out on ice, resuspended in TRIzol (ThermoFisher Scientific) and transferred into bead beating tubes containing 0.5 mm zirconia beads (Biospec). Culture suspensions were bead beaten three times for 30 sec, with cooling on ice between each beating cycle. Samples were then spun down at 13,000 rpm, and the supernatant was transferred to new tubes. Total RNA isolation was performed with the Direct-zol RNA Miniprep Kit (Zymo Research) as per manufacturer’s protocol with on-column DNase treatment. First-strand cDNA synthesis was performed using oligo-dT20 (IDT, Singapore) and the SuperScript III Reverse Transcriptase kit (ThermoFisher Scientific). Quantitative PCR was performed with Luna universal qPCR mastermix (NEB, U.S.A.) as per manufacturer’s protocol on the Applied Biosystem StepOne Plus Real-Time PCR System (ThermoFisher Scientific), using *M. furfur* specific primers designed using PrimerBlast. All primers used in this study were first assessed to have primer efficiency of >90%. List of qPCR primers is as follows:

### Protease activity assay

To determine the extracellular protease activity in each of the culture conditions, cultures grown on 1.5% agar were first removed using a cell scraper. The agar was then sliced and transferred into 20 ml of 50 mM sodium citrate buffer pH 4.2. This was mixed with rotation for 20 min to allow secreted proteases to diffuse out and centrifuged at 7000 rpm. The supernatant was then filtered using a 0.22 µm filter. For cultures in broth or 0.5% media, the entire culture was centrifuged at 5000 rpm for 10 min, the supernatant transferred to a new tube and sterile filtered. All culture supernatants were then frozen at -20°C. For protease assay, the culture supernatant is thawed at 4°C and diluted 50-fold in 50 mM sodium citrate buffer pH 4.2. For protease activity assay using the quenched fluorescent substrates, a final concentration of 20 µM for each substrate was used. Substrates were purchased from CPC Scientific and stored as 1 mM DMSO stocks. The sequence and catalog no. of each substrate is listed below. Fluorescence was monitored at Ex/Em = 330/390 nm using a FlexStation 3 Multimode microplate reader (Molecular Devices) and the activity over the linear range of the enzyme kinetics was determined.

### Aspartyl protease enrichment and identification from culture supernatant

The aspartyl protease enrichment proceeded according to Li et al. [23]. Briefly, washed pepstatin A-agarose beads (Sigma Aldrich) were added to the culture supernatant, incubated at 4°C for 1 h, and washed with 50 mM sodium citrate and 500 mM sodium chloride pH 4.2, followed by 50 mM Tris-HCl pH 5.0 buffer. Elution was performed with 100 mM sodium bicarbonate, 500 mM sodium chloride pH 10.0 and buffer exchanged into 50 mM sodium citrate pH 4.2. The purified proteins were then flash frozen in liquid nitrogen and stored at −80°C. Identification of each protein band by in-gel digestion followed by LC-MS/MS was performed as reported in Li et al. [23].

### Recombinant expression of MfSAP2 in *P. pastoris*

Expression of MfSAP2 was done in *P. pastoris* as previously described for MgSAP1 [23]. Briefly, MfSAP2 was codon-optimized, synthesized, and cloned into pPicZ (Genscript). The plasmid was transformed into *P. pastoris* strain GS115 following the condensed transformation protocol by Lin-Cereghino et al. [31] and plated on yeast extract-peptone-dextrose (YPD) agar containing 100 µg/ml zeocin and grown at 30°C for 72 h. Colonies from the plate were used to start 4 ml starter cultures grown in YPD, which were then shaken at 250 rpm, 30°C for 72 h. 1 l baffled Erlenmeyer flasks containing 200 ml of buffered minimal methanol-complex media (BMMY) were inoculated with the starter cultures, and shaken at 250 rpm, 30°C. To induce expression of MfSAP2, 2 ml methanol was added every 12 h for 72 h. Cultures were harvested by centrifugation at 3000 ***g*** for 10 min at 4°C, and supernatants were sterile filtered and stored at −20°C.

### Ion-exchange (IEX) and size exclusion chromatography (SEC) purification of MfSAP2

All FPLC purification steps were performed on the ÄKTA start protein purification system (Cytiva). The stored *P. pastoris* supernatant containing rMfSAP2 was thawed, syringe filtered with 0.22 µm filters, and buffer exchanged into 20 mM sodium citrate pH 3.5.

Ion-exchange chromatography was performed on the HiTrap SP HP 5 ml cation exchange column (Cytiva), at a flow rate of 5 mL/min. The supernatant was loaded onto the column and washed with 25 ml of 95% buffer A and 5% buffer B, where buffer A: 20 mM sodium citrate pH 3.5, and buffer B: 20 mM sodium citrate pH 3.5 and 1.0 M NaCl. Proteins were then eluted from the column using a 6 min linear gradient from 5% B to 12% B, followed by a 15 min linear gradient from 12% B to 80% B. Proteins were collected in 5 ml fractions, and each fraction was assayed for protease activity with internally quenched fluorogenic peptide S6 as described earlier. The fractions with protease activity against peptide S6 were then pooled, concentrated, and buffer exchanged into SEC buffer containing 150 mM Na_3_PO_4_ and 300 mM NaCl buffer pH 7.2, and stored at −80˚C in preparation for size exclusion chromatography.

Size exclusion chromatography was performed on the HiPrep 16/60 Sephacryl S-100 HR column (Cytiva), at a flow rate of 0.5 ml/min. 1 ml of the buffer-exchanged eluate from the previous IEX step was loaded onto the column, followed by isocratic elution with 120 ml of SEC buffer, collected in 5 ml fractions, and assayed for protease activity against peptide S6. Fractions with protease activity were pooled and buffer exchanged into 50 mM Tris pH 4, then concentrated and stored at −80°C. The purified fractions were not pooled based on the UV absorbance at 280 nm due to low protein yield resulting in a weak UV trace.

### MfSAP2 substrate profiling by mass spectrometry

A defined library of 228 14-mer peptides that represents 2964 unique potential cleavage was used as a substrate pool for uncovering the substrate specificity of MfSAP2 (26; Rohweder et al., 2023). MfSAP2 was mixed with an equimolar mixture of all 228 peptidases in 50 mM sodium citrate/citric acid pH 4.2 such that the final concentration of enzyme was 0.1 µg/ml and each peptide was 0.5 μM. Samples were prepared in quadruplicate reactions, incubated at room temperature, and inactivated by mixing 1:5 in 8 M urea at time intervals of 15 min, 1 h, and 4 h. Quadruplicate negative control samples were prepared by immediately inactivating MfSAP2 with 8 M urea prior to mixing with the peptides. Samples were desalted, dried down, and rehydrated in 0.1% TFA. Each sample was subjected to LC-MS/MS analysis and the raw data files were processed using PEAKS (Rohweder et al., 2023). MS2 data were searched against the 228-member tetradecapeptide library sequences, and a decoy search was conducted with sequences in reverse order. A precursor tolerance of 20 ppm and 0.01 Da specified. Data were filtered to 1% peptide and protein level false discovery rates with the target-decoy strategy. Peptides were quantified with label-free quantification, and data were normalized and filtered by 0.5 peptide quality. Using R scripts, outliers from replicates were removed by Dixon’s Q testing. Missing and zero values are imputed with random normally distributed numbers in the range of the average of smallest 5% of the data ± SD. Analysis of variance testing was performed for peptide data of control and 60 min incubation conditions; those with *P*<0.05 were considered for further analysis. Criteria for cleaved peptide products were those with intensity scores of eight-fold or more in the 4 h dataset relative to the negative control with *P*<0.05 by the two-tailed homoscedastic *t*-test.

### *In vitro* extracellular matrix (ECM) protein degradation assays

ECM proteins were purchased from commercial sources as follows: fibronectin (Sigma Aldrich #F0895), rat tail collagen type I (BD Bioscience, #354236), collagen IV from human placenta (Sigma Aldrich #C5533), keratin from human epidermis (Sigma Aldrich #K0253), laminin from human fibroblasts (Sigma Aldrich #L4544), vitronectin from human plasma (Sigma Aldrich #5051) and human thrombospondin-1 (Sigma Aldrich #ECM002). Degradation of the proteins was assessed by incubating each protein at varying substrate to enzyme ratios of enriched MfSAP2 for 4 h at 34°C in 50 mM sodium citrate buffer, pH 4.2. 4X Laemmli sample buffer (Bio-Rad) with 10% β-mercaptoethanol (Sigma-Aldrich) was added at the endpoint, boiled for 5 mins, and loaded onto 12% TGX FastCast SDS-PAGE gels (Bio-Rad). The gels were stained with Bio-Safe Coomassie G-250 (Bio-Rad) and imaged on the ChemiDoc MP (Bio-Rad).

For assessment of skin epidermal proteins degradation, whole human skin was obtained from surgical discard under IRB B-16–135E. Human epidermis was peeled off from the skin after overnight incubation in 1M sodium chloride solution and frozen at -80°C. The harvested epidermis was then lysed in 1 ml of SDS-Urea buffer (1% SDS, 8 M urea, 10 mM Tris pH 7.4, 140 mM NaCl, 5 mM EDTA, 2 mM EGTA) containing cOmplete EDTA-free protease inhibitor cocktail (Roche), sonicated in an ultrasonication ice bath at 4°C for 20 min, and then centrifuged at 15,000 rpm for 40 min at room temperature to remove the insoluble material. The soluble proteins were then precipitated by diluting the supernatant by six times in precipitation solution (EtOH: acetone: acetic acid = 50%: 50%: 0.1%) and kept at −20°C overnight. The next day, the mixture was centrifuged at 15,000 rpm for 40 min at 4°C to collect the precipitate. The precipitated protein was then washed with the pre-cooled acetone once and dried in a Genevac EZ-2 centrifugal evaporator (Scientific Products).

To investigate the degradation of the skin epidermal protein, 20 µg of skin epidermal protein and a series ratio of purified MfSAP2 were incubated for 4 h at 34°C in 50 mM sodium citrate buffer pH 4.2. 4X Laemmli sample buffer (Bio-Rad) was added at the endpoint and boiled for 5 min. Proteins were resolved on 12% TGX FastCast SDS-PAGE gels (Bio-Rad) and transferred at 60 V for 70 min onto PVDF membranes (Bio-Rad) for immunoblotting.

The transferred blots were blocked with 5% milk in 1 x Tris buffered saline with 0.1% Tween 20 (TBST) for 1 h at room temperature and stained at 4°C overnight with the primary antibody. Following a wash with TBST, the membrane was incubated with the appropriate secondary antibody for 45 min at room temperature. ECL western blotting substrate (Promega) was added as per manufacturer’s protocol and the blot was visualized on the iBright FL1500 Imaging System (Invitrogen). After imaging, the membranes were stripped by shaking with 20 ml of stripping solution containing 62.5 mM Tris pH 6.8, 1% SDS, and 0.8% β-mercaptoethanol for 45 min at room temperature. The stripped membrane was then washed 3 times with fresh TBST before reprobing with the next pair of primary and secondary antibodies.

### Generation and treatment of 3D human skin equivalents

3D human skin equivalents were generated as previously described in Robinson et al*.* [32]*.* Briefly, on day 1, a layer of rat tail type 1 collagen matrix (containing human fibroblasts) was deposited onto polyethylene terephthalate membranes (Falcon) to polymerize for 24 h. On day 2, human keratinocytes were seeded on the matrix and incubated for 24 h before the culture was raised to the air-liquid interface on day 3 and fed with media to induce epidermal differentiation. 3D skin equivalents were differentiated for a total of 13 days and treated with the stated concentrations of MfSAP2 protein dissolved in 50 mM Tris pH 5.0 and 20% Pluronic F-127 (Sigma-Aldrich) gel for 24 h and harvested for histology analyses.

### Histology

Heat-induced antigen retrieval (HIER) was performed on deparaffinized skin tissue sections under pressure at 121°C, followed by 30 min blocking in 10% donkey serum (Dako). Tissue sections were incubated overnight with anti-corneodesmosin antibody (R&D Systems) at 4°C in a humidified chamber. Anti-sheep IgG HRP-conjugated secondary antibody (R&D Systems) was applied for 30 min at room temperature, and peroxidase activity was detected using 3,3′-diaminobenzidine tetrahydrochloride substrate (Dako). Images were acquired using a Nikon Ni-E microscope.

## Supplementary material

online supplementary material 1.

online supplementary material 2.

## Data Availability

All data and reagents are available from the authors upon request.

## Open Access

This article has been published open access under our Subscribe to Open programme, made possible through the support of our subscribing institutions, learn more here: https://portlandpress.com/pages/open_access_options_and_prices#conditional

## Abbreviations

CDSN: corneodesmosin
MM: minimal culture medium
PV: pityriasis versicolor
SAP: secreted aspartyl protease
SC: stratum corneum
mLNA: media containing olive oil
