# Supplementary material for: Aspartyl protease MfSAP2 is a key virulence factor in mycelial form of skin fungi Malassezia furfur
Source: Biochem J. 2025 Dec 24;483(1):BCJ20253109. doi: 10.1042/BCJ20253109 (PMC12794316; doi:10.1042/BCJ20253109)

SI1

A

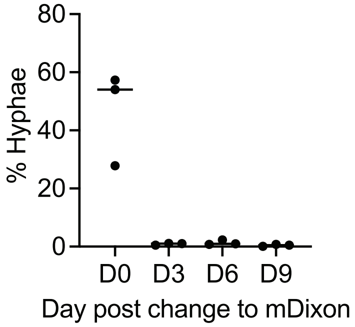

B

Condition	Innoculum	CBS7019 condition media	Fresh media	Hyphae?
1	CBS14141	None	12 mL MM	No
2	CBS14141	None	12 mL MM+	No
3	CBS14141	6 mL MM broth	6 mL MM	No
4	CBS14141	6 mL MM+ broth	6 mL MM+	No
5	CBS14141	6 mL MM+ 0.5% agar	6 mL MM+	No
6	CBS14141	3 mL MM broth	9 mL MM	No
7	CBS14141	3 mL MM+ broth	9 mL MM+	No

Condition 2

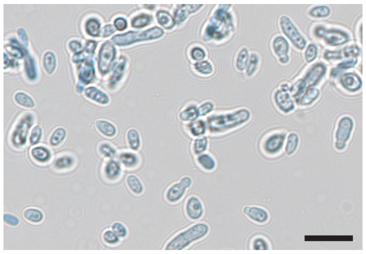

Condition 4

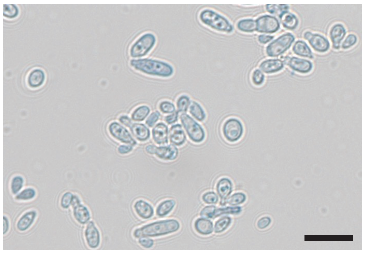

Figure S2

a

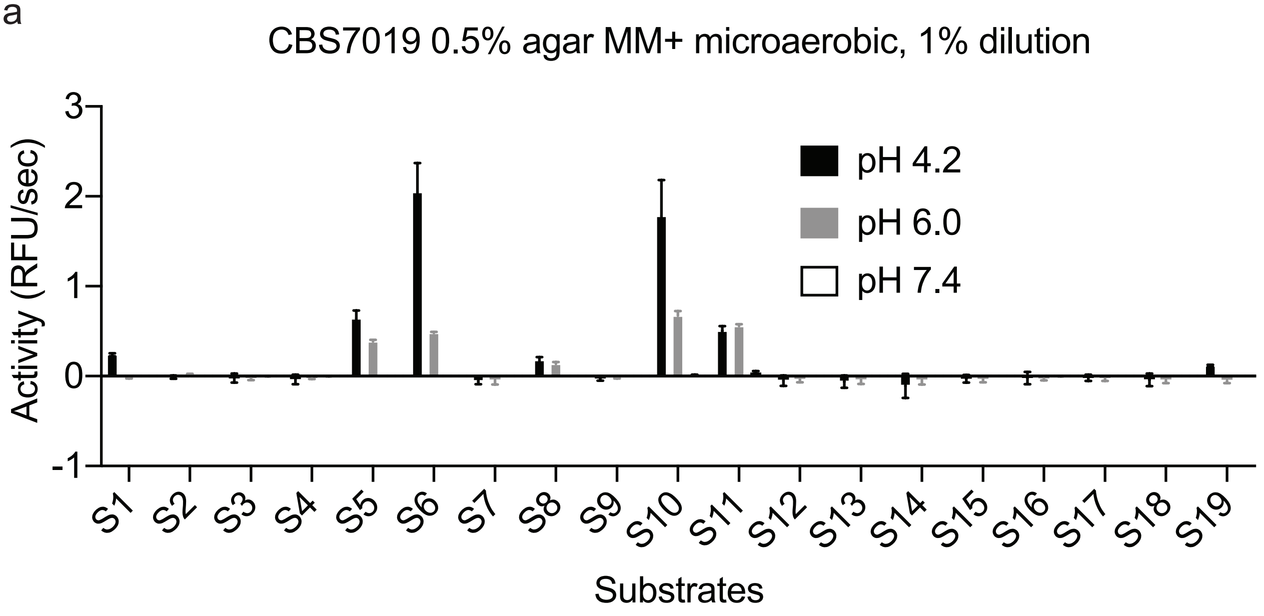

CBS7019 0.5% agar MM+ microaerobic, 1% dilution
Activity (RFU/sec)
3
2
1
0
-1
pH 4.2
pH 6.0
pH 7.4
S1 S2 S3 S4 S5 S6 S7 S8 S9 S10 S11 S12 S13 S14 S15 S16 S17 S18 S19
Substrates

b

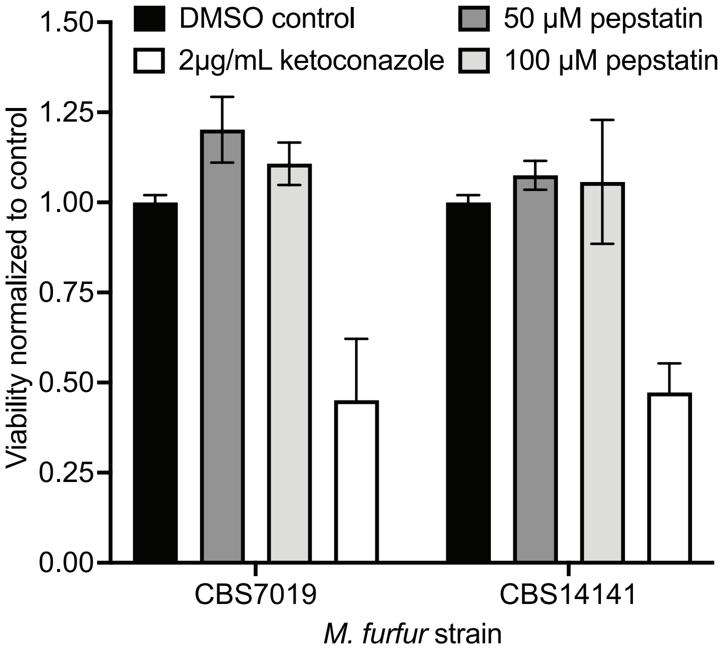

DMSO control
50 µM pepstatin
2µg/mL ketoconazole
100 µM pepstatin
Viability normalized to control
1.50
1.25
1.00
0.75
0.50
0.25
0.00
CBS7019
CBS14141
M. furfur strain

Figure S3

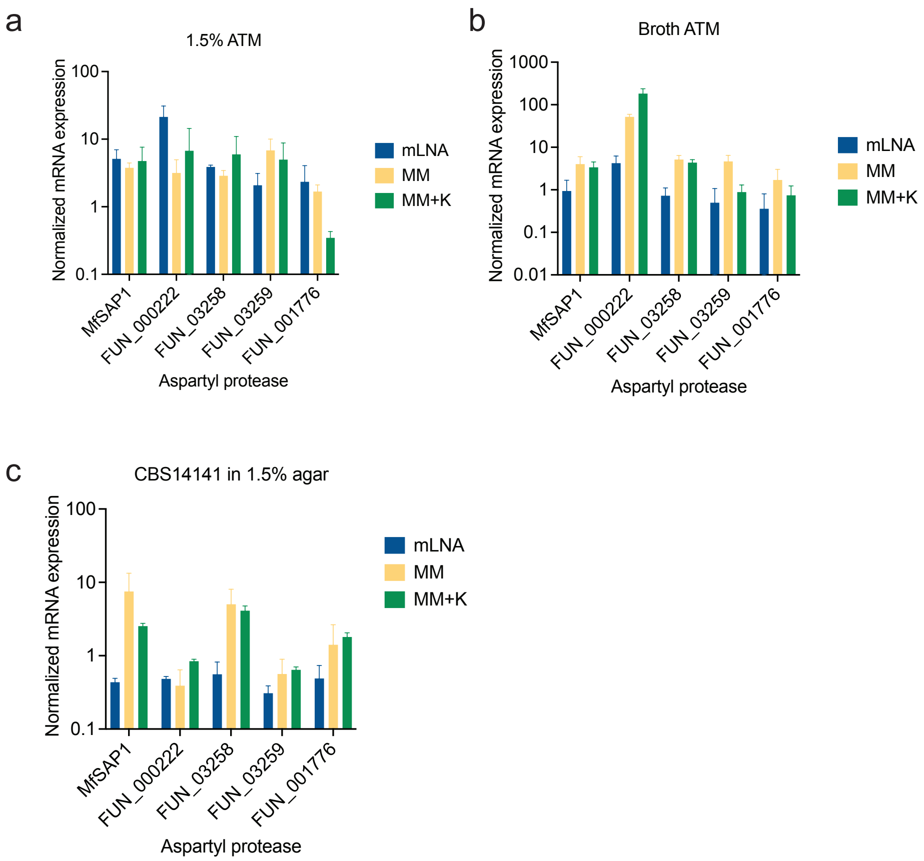
a
1.5% ATM
Normalized mRNA expression
100
10
1
0.1
MfSAP1
FUN_000222
FUN_03258
FUN_03259
FUN_001776
Aspartyl protease
mLNA
MM
MM+K
b
Broth ATM
Normalized mRNA expression
1000
100
10
1
0.1
0.01
MfSAP1
FUN_000222
FUN_03258
FUN_03259
FUN_001776
Aspartyl protease
mLNA
MM
MM+K
c
CBS14141 in 1.5% agar
Normalized mRNA expression
100
10
1
0.1
MfSAP1
FUN_000222
FUN_03258
FUN_03259
FUN_001776
Aspartyl protease
mLNA
MM
MM+K

Figure S4

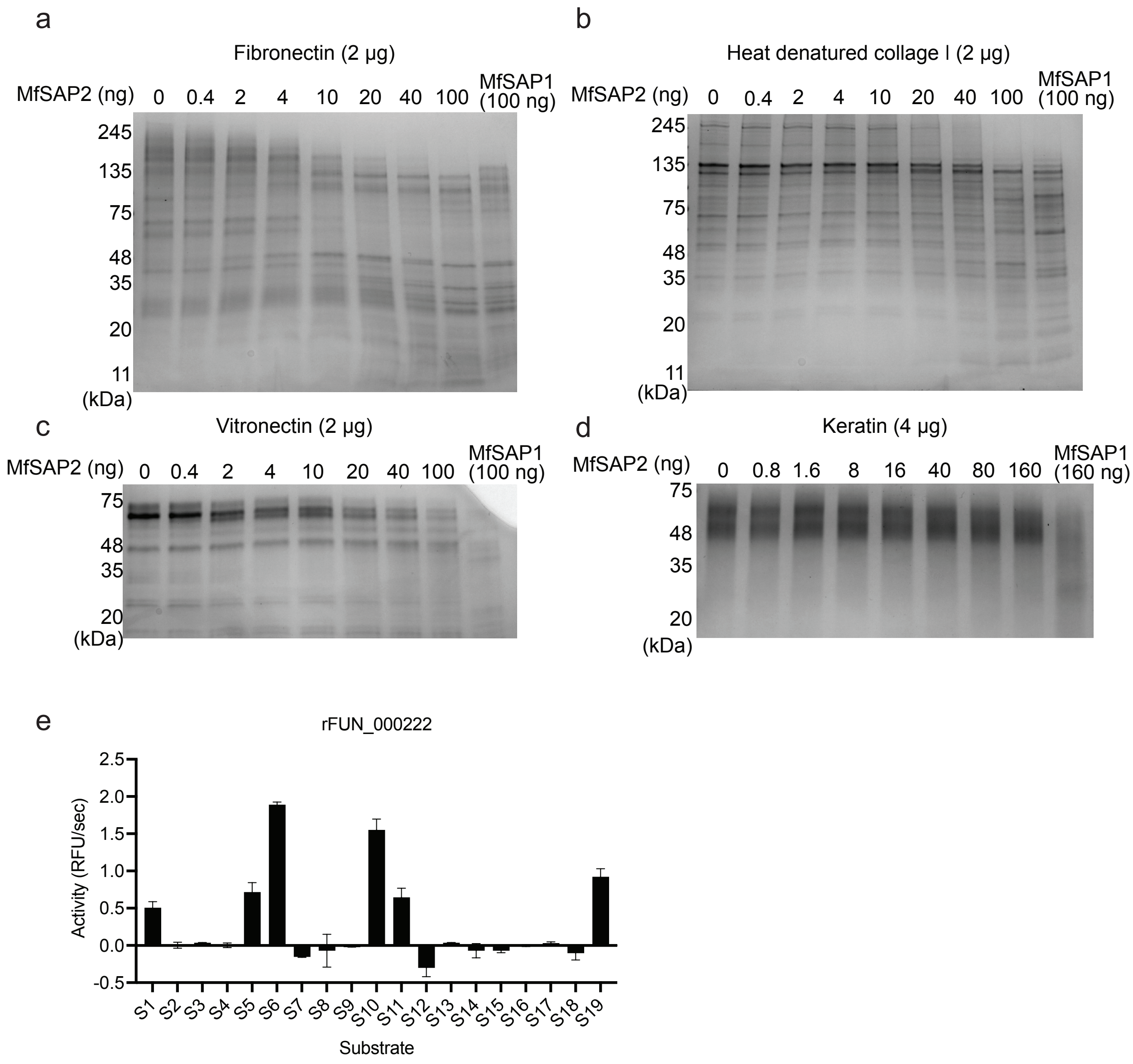

a
Fibronectin (2 µg)
MfSAP2 (ng) 0 0.4 2 4 10 20 40 100 MfSAP1 (100 ng)
245
135
75
48
35
20
11
(kDa)
b
Heat denatured collage I (2 µg)
MfSAP2 (ng) 0 0.4 2 4 10 20 40 100 MfSAP1 (100 ng)
245
135
75
48
35
20
11
(kDa)
c
Vitronectin (2 µg)
MfSAP2 (ng) 0 0.4 2 4 10 20 40 100 MfSAP1 (100 ng)
75
48
35
20
(kDa)
d
Keratin (4 µg)
MfSAP2 (ng) 0 0.8 1.6 8 16 40 80 160 MfSAP1 (160 ng)
75
48
35
20
(kDa)
e
rFUN_000222
Activity (RFU/sec)
2.5
2.0
1.5
1.0
0.5
0.0
-0.5
S1 S2 S3 S4 S5 S6 S7 S8 S9 S10 S11 S12 S13 S14 S15 S16 S17 S18 S19
Substrate

SI5- Supplemental figure for Western Blot images in Figure 5

Figure 5D Desmoglein 1

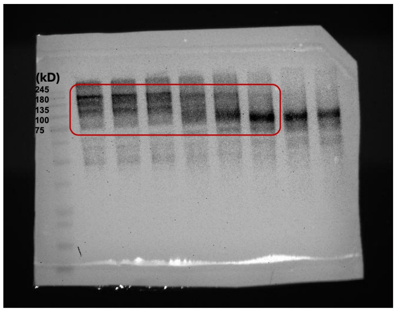

Figure 5D β-actin

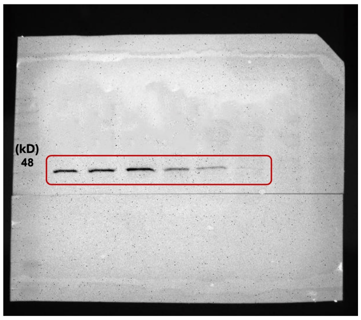

Figure 5D Desmocollin

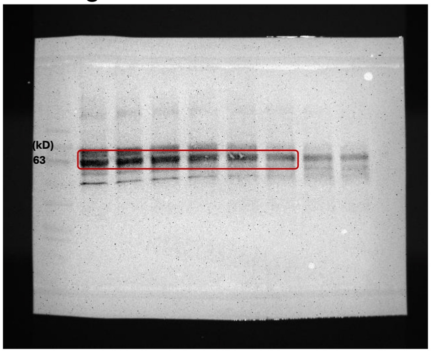

Figure 5E Corneodesmosin

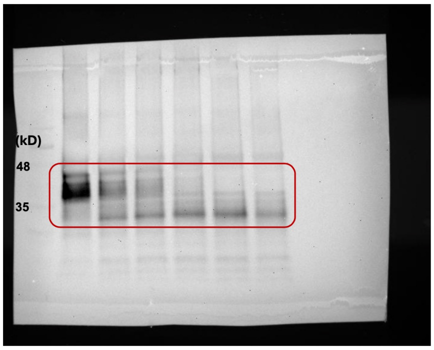

Figure 5D Loricrin

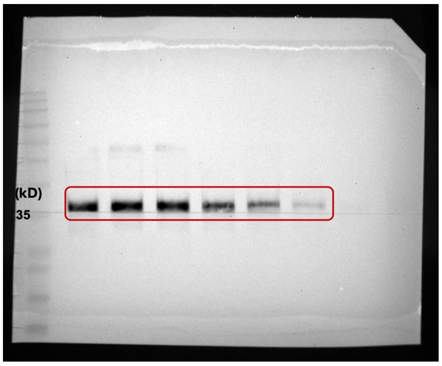

Figure 5E β-actin

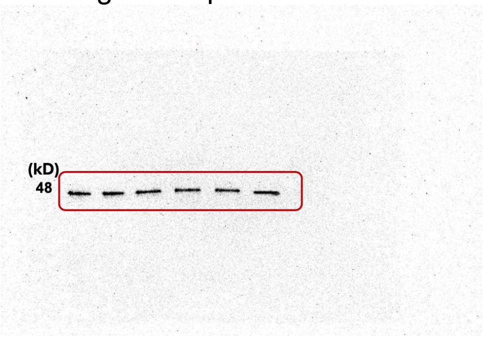

Supplement: online supplementary material 1. [file bcj-483-1-BCJ20253109-s001.pdf]
