# Supplementary material for: Aspartyl protease MfSAP2 is a key virulence factor in mycelial form of skin fungi Malassezia furfur
Source: Biochem J. 2025 Dec 24;483(1):BCJ20253109. doi: 10.1042/BCJ20253109 (PMC12794316; doi:10.1042/BCJ20253109)
Supplement: online supplementary material 2. [file bcj-483-1-BCJ20253109-s002.docx]

**Table S1: List and sequences of qPCR primers used for transcription analysis.**

| **Gene** | **Forward Primer** | **Reverse Primer** |
| --- | --- | --- |
| MfSAP1 | TCGGTCACTCGTTCACCAAG | GAGGCTCTCAAAGAACGGCT |
| MfSAP2 | ACAGACGTTTGGCATCGACT | CGAAGTCTGGAATGGCTGGT |
| MfSAP3 | ATACGTCGCTGTATGAGGGC | TTGGGTATCACGGGGGAGAT |
| MfSAP4 | AAGAATGCGAAGAAGCACGC | CAGCACATTCACGGTTGTCG |
| MfSAP5 | CTCCTACTCGTGCTGGCTTC | TGCATCCACTGCAATAACGC |
| *M. furfur* actin | CGGCAACATTGTCATGTCGG | GCAAGGATCGAACCACCGAT |

**Table S2: List of quenched fluorescent substrates used to determine protease activity.**

| **Substrate code** | **CPC Catalogue No.** | **Sequence** |
| --- | --- | --- |
| S1 | AMYD109 | Mca-SEVNLDAEFRK(DNP)-RR-NH2 |
| S2 | AMYD111 | Mca-RPPGFSAFK(DNP)-NH2 |
| S3 | AMYD112 | Mca-HQKLVFFAK(DNP)-NH2 |
| S4 | AMYD114 | Mca-EVKMDAEFK(DNP)-NH2 |
| S5 | MMPS024 | Mca-RPKPYANvaWMK(DNP)-NH2 |
| S6 | SUBS017 | Mca-GKPILFFRLK(DNP)-r-NH2 |
| S7 | CAPS060 | Mca-VDQMDGWK-(DNP)-NH_2_ |
| S8 | MMPS009 | Mca-PLGL-Dap(Dnp)-AR-NH_2_ |
| S9 | MMPS016 | Mca-P-Cha-G-Nva-H-A-Dap(DNP)-NH_2_ |
| S10 | MMPS026 | Mca-RPKPVENva-WRK(DNP)-NH_2_ |
| S11 | MMPS029 | Mca-KPLGL-Dap(Dnp)-AR-NH_2_ |
| S12 | CASP027 | Mca-YVADAP-Lys(DNP)-OH |
| S13 | CASP028 | Mca-VDQVDGW-Lys(Dnp)-NH_2_ |
| S14 | CASP059 | Mca-DEVDAP-Lys(Dnp)-OH |
| S15 | CASP068 | Mca-LEVDGWK(DNP)-NH_2_ |
| S16 | AMYD103 | Mca-VNLDAEF-Lys(Dnp)-NH_2_ |
| S17 | AMYD105 | Mca-SEVNLDAE-Dap(Dnp)-NH_2_ |
| S18 | AMYD108 | Mca-SEVNLDAEF-K(DNP)-NH_2_ |
| S19 | AMYD110 | Mca-SEVKMDAEFR-K(DNP)-RR-NH_2_ |

**Table S3:List of primary and secondary antibodies used for Western blot.**

| Primary antibodies | | | Secondary antibodies | |
| --- | --- | --- | --- | --- |
| Target protein | Product info | Reactive species | Product info |  |
| Desmoglein 1 | Abcam ab12077 | Mouse | Anti-Mouse lgG (H+L), HRP conjugate | Promega W4021 |
| Desmocollin | Meridian Life Science H44951M |  |  |  |
| Actin | Santa Cruz Biotechnology sc-517582 |  |  |  |
| Loricrin | Abcam ab176322 | Rabbit | Anti-Rabbit lgG (H+L), HRP conjugate | Promega W4011 |
| Corneodesmosin | R&D SYSTEMS AF5725 | Sheep | Donkey Anti-Sheep lgG (H+L) HRP conjugate | Life Technologies^TM^ A16041 |
